# Principles of Medicine

**Published:** 1843-12-09

**Authors:** 


### BIBLIOGRAPHICAL NOTICES.

*Principles of Medicine. Comprising General Pathology, Therapeutics, and a Brief General View of Etiology, Nosology, Semeiology, Diagnosis, and Prognosis.* By Charles J. B. Williams, M. D., F. R. S., Professor of the Principles and Practice of Medicine, etc. etc. etc. *With Additions and Notes.* By Meredith Clymer, M. D., Lecturer on the Institutes of Medicine, Physician to the Philadelphia Hospital, Fellow of the College of Physicians, etc. etc. Philadelphia: Lea & Blanchard. 1844. 8vo, pp. 383.

The author offers, in his preface, as an apology for for the present publication, the actual want of a work on General Pathology, and its application to Practical Medicine; a want which, in common with most teachers of the practice of medicine, he has himself experienced. An able cotemporary has recently thus defined the scope of a treatise on General Pathology: "The legitimate object of a work on General Pathology is the study of diseases in the abstract, and in that which they have in common. It should serve as a complement to special and descriptive pathology, just as general anatomy does to descriptive anatomy. It should comprise all that is most simple and most elevated in science; on the one hand, the definition of the terms, and the description of the phenomena of diseases; on the other hand, the discussion of all the fundamental questions, and the exposition of the general principles which should serve to guide the physician in the arduous exercise of a profession so intimately connected with the dearest interests of humanity. General Pathology, in fact, includes within itself the humblest elements, and the most sublime philosophy of medicine." In France the works of Chomel and Dubois stand deservedly high, especially the former and that of Neumann in Germany holds justly an elevated rank. The "Principles" of Dr. Williams is, we think, entitled to assume a station along with these. It is without a competitor in our language, and fills most successfully a decided gap in our medical literature. The actual state of our science is very fairly represented, and besides a free appropriation of the writings of his contemporaries, the author has drawn largely on his own experience; "a continual observation of disease for the last twenty years in hospital and private practice," affording him abundant opportunity for its accumulation. The following sound remarks we extract from the preface:—

"It seems quite extraordinary, that notwithstanding the recent rapid improvements and comparative perfection of the contributory sciences, practical medicine should still halt in the domain of empiricism. A chief reason for the anomaly seems to be that science and practice have rarely been pursued by the same parties. Scientific men are not and cannot be practical, because they have had no experience; and practitioners know little of science; and therefore derive little good from it. Instead of working together, these parties are at issue with each other. But it is high time to put an end to this feud. Philosophers must descend from their transcendental positions, to consider the details of practice and purposes of utility. Those who would be practitioners, must gain from science that knowledge and that method which render experience instructive and useful."—(*Preface*, p. iv.)

An Introduction, on "the Need of the Study of General Pathology as the Foundation of the Study of Practical Medicine," embodies much truth. The present low condition of medical science with the public is referable, Dr. Williams thinks, to the faulty mode of teaching Practical Medicine, an opinion in which we fully concur. Whilst the condition of the contributory sciences is precise and definite, that of the practice of medicine is loose and uncertain. "To the public it appears altogether vague, without any acknowledged principles."—(p. 13.) The result is, the triumph of quackery; and most appalling, indeed, is its progress in both continents. The public have no faith in legitimate medicine, because it so frequently fails to accomplish what it promises, and they have a ready credence for any novelty. "The public show their ignorance by such credulity; but they show also the want of something plain and trustworthy in regular medicine. The public will not believe that the secret of the art is with a faculty which professes to follow experience only." The quack can appeal to his experience, and does so loudly, if not triumphantly. The consequence is not a determination on the part of the profession to contribute to the elevation of practical medicine in the eye of the community, by the cultivation of general principles, and their application to the legitimate objects of our art, but too often a recourse to the very means which are undermining medicine—the opposition of quackery by quackery—the application of the homœpathic maxim *similia similibus curantur*, and with about the same amount of success—the infusion of a spirit of charlatanism into the profession at large.

When a few weeks ago we looked at that body of young men, assembled from every section of our country, for the attainment of a knowledge of practical medicine, enthusiastic and ardent in their object, we could not help thinking that if quackery flourish in the land there must be something wrong in those who have the instruction and guidance of such excellent and malleable materials. If such are properly taught the principles of medicine, and a proper spirit is infused into them, it is impossible that medical charlatanism can survive; the extended and united influence of such bodies of men as our medical schools annually send forth, must necessarily soon exterminate quackery, if they acquire in the course of their education an abiding reverence for their art. The evils of quackery are due, in a great measure, we feel assured, to the imperfect system of medical education; to the selfiish principle of aggrandisement which prevails amongst teachers and their institutions; and to the neglect, if not contempt of the sacred duty imposed upon them. "The practice of medicine, as taught, is an enormous mass of dry detail: its science mere glimpses into an unknown land; its rules irregular tracks through a wilderness of confusion." A distaste is thus acquired for the most useful and necessary branch, and the result is disappointment, with a mistrust and contempt for principles, and the subsequent adoption of a pure empiricism.

In the first chapter, Etiology is treated of in some detail, and the cause of diseases investigated with considerable acumen. The second chapter treats of Pathology Proper, or Pathogeny. The disorders of the various Primary Elements are examined at length. The sections on the Blood are very complete, and exhibit the actual state of our knowledge on this important point fairly and cleverly. The Proximate Elements of Disease are next considered; these embrace Anemia, Hyperemia, Congestion, and Inflammation. The section on Inflammation is especially worthy of attention: many of the views are original and of a highly practical bearing. Chapter VI. treats of Structural Diseases, or Diseases of Nutrition; as Atrophy, Hypertophy, Softening, Textural Deposits, Non-Malignant, and Malignant Growths. The Classification, Symptoms, and Distinction of Diseases, including Nosology, Semeiology and Diagnosis, are comprised under chapter V. Prognosis occupies the whole of chapter VI. and is treated of at length, and includes the various modes of death, and their operation on the blood. Chapter VII, and the concluding one, treats of Prophylaxis, and Hygiene.

Some parts of the work were stated by the author, in his preface, to be incomplete, owing to want of time "To supply the omissions has been the endeavour of the American Editor. The chief additions will be found in the sections on the Blood, on Diagnosis and on Prognosis. A chapter on Fever and Hygiene have also been added."—(*Preface*, p. v.)

